# Three-Dimensional Printing of Ultrasoft Silicone with a Functional Stiffness Gradient

**DOI:** 10.1089/3dp.2022.0218

**Published:** 2024-04-16

**Authors:** Clayton A. Young, MeiLi O'Bannon, Scott L. Thomson

**Affiliations:** Department of Mechanical Engineering, Brigham Young University, Provo, Utah, USA.

**Keywords:** silicone 3D printing, functionally graded 3D printing, ultrasoft 3D printing, functional stiffness gradient, multi-material printing, biomechanical modeling

## Abstract

A methodology for three-dimensionally printing ultrasoft silicone with a functional stiffness gradient is presented. Ultraviolet-cure silicone was deposited via two independently controlled extruders into a thixotropic, gel-like, silicone oil-based support matrix. Each extruder contained a different liquid silicone formulation. The extrusion rates were independently varied during printing such that the combined selectively deposited material contained different ratios of the two silicones, resulting in localized control of material stiffness. Tests to validate the process are reported, including tensile testing of homogeneous cubic specimens to quantify the range of material stiffness that could be printed, indentation testing of cuboid specimens to characterize printed stiffness gradients, and vibratory testing of synthetic multilayer vocal fold (VF) models to demonstrate that the method may be applied to the fabrication of biomechanical models for voice production research. The cubic specimens exhibited linear stress–strain data with tensile elasticity modulus values between 1.11 and 27.1 kPa, more than a factor of 20 in stiffness variation. The cuboid specimens exhibited material variations that were visually recognizable and quantifiable via indentation testing. The VF models withstood rigorous phonatory flow-induced vibration and exhibited vibratory characteristics comparable to those of previous models. Overall, while process refinements are needed, the results of these tests demonstrate the ability to print ultrasoft silicone with stiffness gradients.


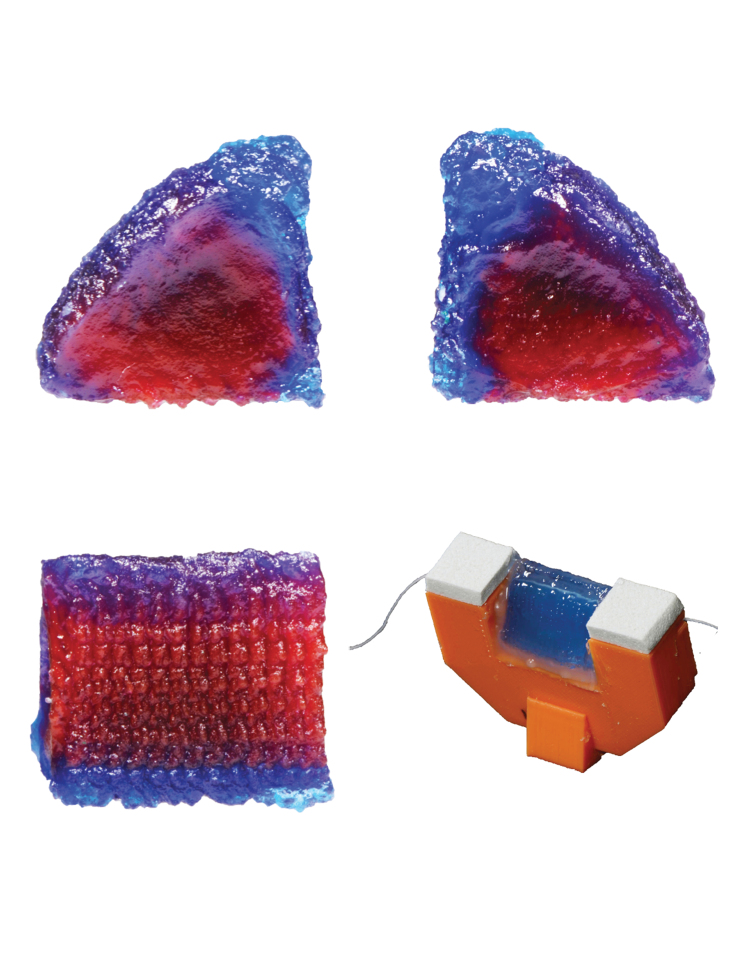


## Introduction

**FIG. 1.**
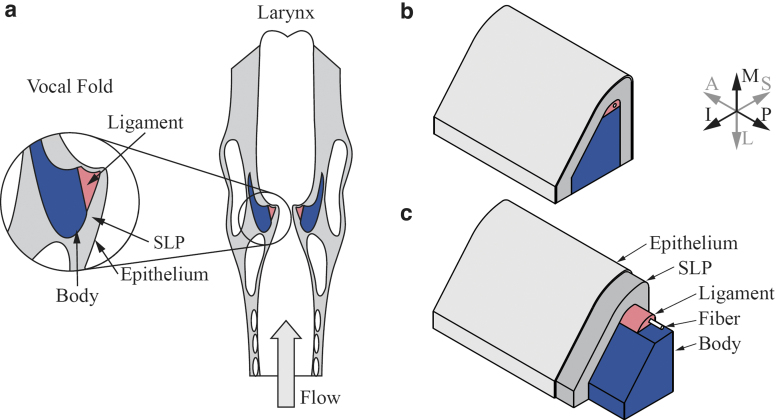
**(a)** Illustration of frontal view of the human larynx showing VF tissue layers. **(b)** EPI VF model with **(c)** cutaway view of corresponding material layers. Model measures ∼17 mm in the A–P direction. Anatomical orientations are anterior (A), posterior (P), inferior (I), superior (S), medial (M), and lateral (L). Illustrations used and modified with permission as follows: **(a)** Vaterlaus^37^ and Greenwood,^28^ **(b, c)** Greenwood and Thomson.^26^ EPI, epithelium; VF, vocal fold.

Sound for voiced human speech is produced by the flow-induced vibration of the vocal folds (VFs). During voicing, the VFs undergo large amplitude oscillation at relatively high frequencies. Synthetic self-oscillating VF models are used to study various physical aspects of human voice production, such as vibration frequency, onset pressure (the minimum lung pressure required to initiate vibration), and glottal width amplitude.^1^ The human VFs are composed of different types of tissues, and synthetic VF models are typically fabricated with distinct layers of silicone of differing stiffness to mimic the human VF tissue structure. For example, the so-called “epithelium” (EPI) model^2,3^ includes the body, ligament, superficial lamina propria (SLP), and epithelium layers, each with different stiffness (Fig. 1). Some of the silicone layers are exceedingly soft, with elastic modulus values as low as the order of 10^2^ to 10^3^ Pa. While these models are composed of layers with distinct boundaries, spatial transitions between different tissue types in the human VFs may be more gradual.

The most common synthetic VF model fabrication process involves sequentially casting different layers.^2,3^ This fabrication process is a viable approach to creating models that exhibit vibratory characteristics that are comparable to those of human VFs but is also limited by high failure rate, relatively long fabrication time, and distinct layers. Consequently, alternative fabrication processes based on silicone three-dimensional (3D) printing have been explored.

In non-VF-related applications, single-material silicone 3D printing has been achieved using approaches, such as digital light processing,^4–7^ inkjet printing,^8^ stereolithography,^9^ embedded printing,^10,11^ and direct ink writing.^12–17^ Applications of single-material silicone 3D printing include soft robotics,^4,14,17,18^ flexible electronics,^8,12,16,19^ and biomedical implants.^11,15,20,21^ Several of these approaches may be viable for fabricating single-material VF models; however, further development is needed to print VF models with spatial variations in stiffness. Multi-material 3D printing approaches have been explored^9,17,18,21–23^ but have either not shown the ability to print a true material gradient or the print material has not been suitably soft for VF models.

In VF-related applications, Romero *et al.*^24^ built upon the method of O'Bryan *et al.*^10^ to fabricate VF models. An ultraviolet (UV)-curable liquid silicone was extruded into a sacrificial gel-like support matrix that held the deposited silicone in place until it could be cured. This method was successfully used to print self-oscillating VF models; however, small voids of uncured support matrix remained inside the VF models. To overcome this limitation, Greenwood *et al.*^25^ developed a silicone oil-based support matrix that cured in the immediate vicinity of the printed silicone to create void-less prints. Greenwood and Thomson^26^ developed a hybrid 3D printing/casting process to fabricate multi-layer VF models by using a VF-shaped mold filled with a curable support matrix and sequentially 3D printing interior VF layers of different silicone stiffness into the support matrix before it cured. This latter concept introduced a potential alternative to traditional casting but was limited by fixed outer geometry and difficulties of demolding exceedingly soft silicone.

In this article, the methodology for creating ultrasoft silicone prints with functional stiffness gradients is presented. Results of tests showing the range of modulus values capable of being printed are reported. Localized control over stiffness variations is demonstrated. Geometric and vibratory data from multi-layer printed VF models are provided, showing the ability to print models that withstand rigorous flow-induced vibration and that exhibit vibratory characteristics comparable to those of previous VF models. Finally, suggestions for future work are proposed.

## Materials and Methods

### Overview

Printing of silicone parts with spatial stiffness variations was accomplished by depositing UV-cure silicone via two independently controlled extruders (“A” and “B”) into the thixotropic, gel-like, silicone oil-based support matrix developed by Greenwood *et al.*^25^ Extruders A and B contained uncured liquid silicones that, if allowed to cure, would each cure to a different stiffness. The extrusion rates were independently varied during printing so that the combined deposited material contained different ratios of A and B, and hence different localized cured stiffnesses between those of A and B. The prints were cured, removed from the support matrix, and cleaned. These processes are summarized below; for further details, see the study by Young^27^ and the provided Supplementary Information.

### Printing software and hardware

A multi-section geometric computational model was first created using computer-aided design software, where different sections represented regions of different stiffnesses. A stereolithography (STL) file of each section was imported into custom slicing software (“slicer”), and the material property of each section was assigned a stiffness value. The slicer calculated the ratio of materials from extruders A and B needed for each section and then created g-code to control the 3D printer (described below). The slicer included several adaptations required for depositing liquid silicone into the thixotropic support matrix (Supplementary Information).

**FIG. 2.**
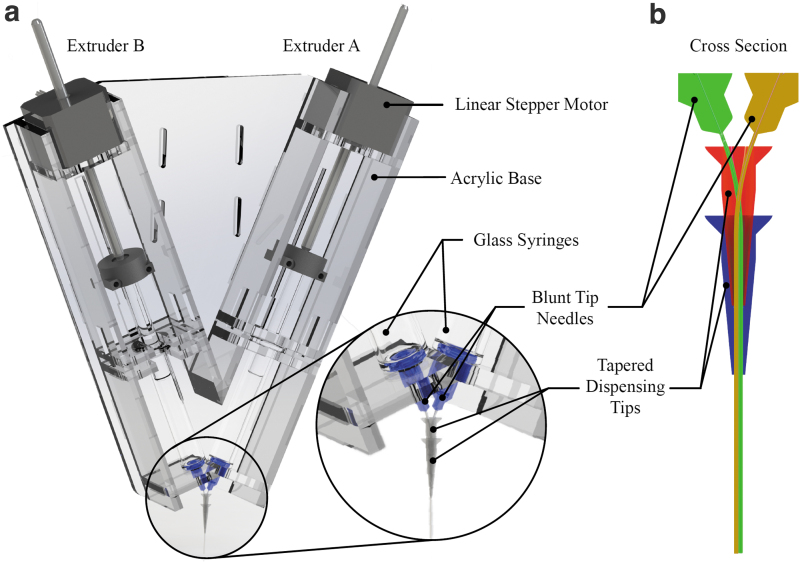
**(a)** Rendering of custom dual extruder and **(b)** illustration of blunt tip needles (*green*, *brown*) guided through two tapered dispensing tips in series (*red*, *blue*).

The 3D printer used by Greenwood *et al.*,^25^ which was a modification of a 7 × 12-inch, 3-axis linear computer numerical control (CNC) Milling Machine (Zen Toolworks, Concord, CA), was retrofitted with the custom dual extruder illustrated in Figure 2. The extruder included two high-torque NEMA 17 motors coupled with high-resolution leadscrews (AMETEK Haydon Kerk Pittman 43J4U-2.33, Waterbury, CT) to control the flow of silicone out of two 5 mL glass syringes (Fortuna Reusable Glass Syringe Luer Tip Model # 7.102–33, Italy). Glass—as opposed to plastic—syringes were chosen to reduce mechanical capacitance (i.e., pressure-induced expansion of syringe walls resulting in excess silicone extrusion while syringe walls return to their original undeformed shapes following stoppage of extrusion motors).

A 1.5-inch long, 27-gauge blunt tip needle (Howard Electronics Blunt Tip, Grand Island, NY) was affixed to each syringe. The needle tips were guided adjacent to one another through two 27-gauge plastic tapered dispensing tips (CML Supply, Lexington, KY) cut to length, as shown in Figure 2. The offset between the needle centers was 0.4 mm and was accounted for in the slicer. Adequate curing of the final prints demonstrated that materials from both syringes were suitably combined when deposited; however, further research is needed to fully characterize mixing of the two materials during printing and to study the effect of the needle offset. The printer hardware was controlled using Mach3 commercial software (Livermore Falls, ME) using the custom slicer-generated g-code.

Print resolution (spacing between centerlines of adjacent paths within a given layer) was a function of CNC resolution (0.0254 mm), extruder stepper motor resolution (0.0015 mm), and blunt tip needle inner diameter (0.21 mm for 27-gauge). The largest of these dimensions is the latter. A print infill of 100% would yield a print resolution of 0.21 mm. However, following Greenwood,^28^ an infill of 60% was used, resulting in an actual print resolution of nominally 0.35 mm (needle inside diameter divided by infill percentage). The layer height was 0.21 mm.

### Printing materials

#### Silicone

The deposited material was the UV-curable silicone elastomer Silopren UV Electro 225 (hereafter referred to as UV Electro; Momentive Performance Materials, Inc., Waterford, NY). Different ratios of Silicone Thinner (Smooth-On, Inc., Macungie, PA) were mixed with UV Electro to achieve the desired material stiffness, that is, adding thinner resulted in lower cured stiffness. The silicone was prepared by first pouring 1 g of thinner into a small mixing container to ensure that the more viscous silicone components that would be subsequently added would not be as susceptible to sticking to the bottom of the container.

Next, the base and catalyst of UV Electro with a 1:20 ratio (base:catalyst by weight) were added, followed by the desired quantity of remaining thinner (discussed below). For visual analysis, pigment (Silc Pig; Smooth-On, Inc.) was occasionally added to provide a color gradient when printing. These components were mixed for 2 min at 2350 rpm using a planetary centrifugal mixer (DAC 150.1 FVZ-K SpeedMixer; FlackTek, Landrum, SC), poured into a syringe, and degassed as needed.

#### Support matrix

The support matrix^25,28^ consisted of 97 parts of silicone thinner and 3 parts of fumed silica (URE-FIL 9; Smooth-On, Inc.) by weight, mixed for 2 min at 2350 rpm in the planetary mixer, degassed for 5 min, and poured into 1 oz. plastic disposable cups for printing.

### Printing procedure

The cup containing support matrix was affixed to the bed of the custom printer. The needle tips were manually positioned into the support matrix, and the print was initiated. Once completed, the cup containing the uncured print and support matrix was placed into a UV curing bed (Dreve Polylux 2000, Unna, Germany) for 5 min. The cured print was then removed from the support matrix, cleaned lightly with a paper towel, agitated in an acetone bath for about 10 s, and blotted with a paper towel to remove excess support matrix.

### Process validation

Tests were performed to validate the printing process, two of which are reported here. The first demonstrated the ability to print a desired stiffness range that is suitable for synthetic VF modeling and the second demonstrated the ability to print variations in stiffness. Additional tests and details are documented in the study by Young.^27^

#### Material stiffness

UV Electro with a 1:2 [(base+catalyst):(thinner)] ratio by weight was placed in extruder A and UV Electro with a 1:12 ratio was placed in extruder B. A computer model of a 10 mm cube was imported into the slicer and assigned a ratio of 1:*N*, where *N* was the desired mixing ratio of thinner (to be controlled by varying the relative extrusion amounts of A and B). Triplicates of cubes with ratios of 1:2 (i.e., printed using only extruder A), 1:4, 1:6, 1:8, 1:10, and 1:12 (i.e., printed using only extruder B) were printed (18 cubes total). The print time was ∼11 min per cube.

Cubes were adhered to acrylic mounting plates using Sil-Poxy (Smooth-On, Inc.) and mounted to an Instron 3342 Universal Testing System (Instron, Norwood, MA) equipped with an Instron 2519-10N 10 N load cell. The tensile test began with 10 preloading cycles from −10% to +25% strain at a rate of 100 mm/min and finished with a final cycle from −10% to +10% strain at 30 mm/min. Force–displacement data were recorded at strain increments of 2.5%, and the tensile modulus for each cube was calculated using a linear fit from 0% to 10% strain of the final cycle data. The preloading cycles were intended to reduce the effects of initial stretching of the material, and the smaller final strain was deemed adequate since modulus values at higher strains were not needed for this study.

#### Spatial stiffness variations

To demonstrate the ability to produce stiffness variations, computer models were created of 10 × 10 × 33 mm rectangular prisms (cuboids) with *n* different equally spaced sections along the 33 mm length. Two models with discontinuous stiffness variations were created, one with *n* = 2 and the other with *n* = 11 (*n* = ∞ would constitute a continuous gradient). These models were imported into the custom slicer, and each section was assigned a stiffness that increased in a stepwise manner from section to section from 1.5 kPa at one end of the cuboid to 12 kPa at the other end (i.e., 1.5 and 12 kPa for the two sections of the *n* = 2 cuboid; and 1.50 kPa, 2.55 kPa, 3.60 kPa, 4.65 kPa, 5.70 kPa, 6.75 kPa, 7.80 kPa, 8.85 kPa, 9.90 kPa, 10.95 kPa, and 12 kPa for the 11 sections of the *n* = 11 cuboid). Two compounds of UV Electro:thinner with nominal stiffnesses of 1.5 and 12 kPa were mixed using ratios that had been predetermined based on the cube test data (results discussed below). Two *n* = 2 cuboids were printed, one with 45° infill-pattern orientation and the other with 90° orientation, as well as one *n* = 11 cuboid (45° orientation).

The stiffness profiles of the finished cuboids were measured using indentation testing. A custom indenter with a 3 mm-diameter flat head was mounted to the Instron load cell. The cuboid was secured to a linear traverse and manually positioned below the indenter head. Tests were performed at lengthwise locations from 0 to 33 mm in steps of 1.27 mm (25 total locations). For each test, the indenter was plunged into the cuboid at a rate of 40 mm/min and returned at the same rate. The stiffness at each location was calculated using a linear fit to the force–displacement data from plunged depths between 0.5 and 1.5 mm.

For comparison with experimental data and to explore the effects of cuboid edges and material interfaces on the stiffness profiles, corresponding finite element (FE) simulations were performed. The model setup is here briefly summarized, with additional details provided in the Supplementary Information. A FE model of the 10 × 10 × 33 mm cuboid was created using ANSYS ADPL. The section materials were modeled using a linear elastic material with a Poisson's ratio of 0.49, the bottom of the cuboid was fixed in all directions, and the indenter was modeled as a 3 mm diameter circle inscribed on the top of the cuboid with the center being located at the desired testing location. The circle was displaced 2 mm into the cuboid over 40 load steps (i.e., 0.05 mm penetration at each load step) and fixed in all other remaining dimensions. Resulting force and displacement data were used to calculate the stiffness.

### VF model printing and testing

**FIG. 3.**
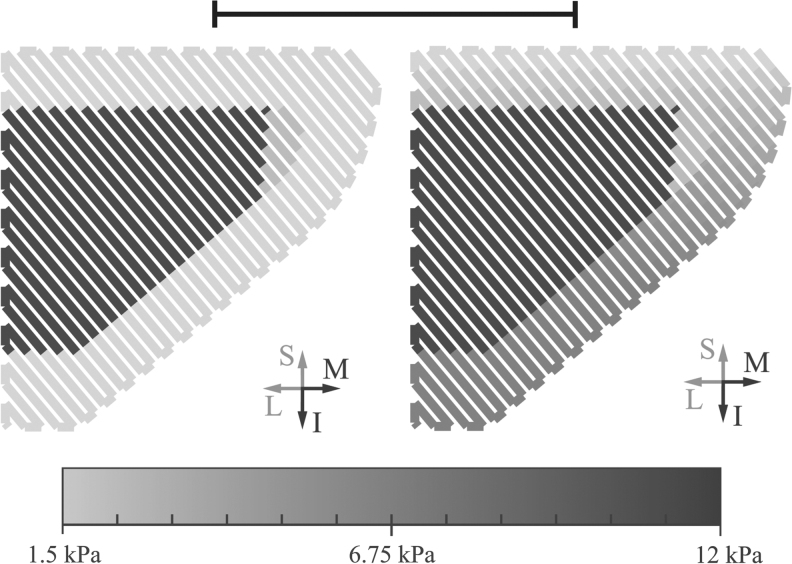
Print paths of EPI (*left*) and VSG (*right*) VF models, with gray scale denoting target material stiffness. Material variation can be observed in the SLP layer of the VSG model. Scale bar (*top*) is ∼10 mm. The models measured 17 mm in the out-of-page (anterior–posterior) dimension. SLP, superficial lamina propria; VSG, vertical stiffness gradient.

Two types of VF models, illustrated in Figure 3, were printed. The first was the so-called EPI model,^2,3^ chosen to demonstrate the ability to print a multilayer VF model. The second, here referred to as the vertical stiffness gradient (VSG) model, was chosen to demonstrate the ability to fabricate VF models with a material gradient that would be difficult to cast. One example of this is the inferior–superior VSG that has been previously studied.^29–31^ The VSG model in this study was a combination of the EPI model and the model created by Geng *et al.*^30^ The VSG model geometric and stiffness characteristics are shown in Figure 3, and the corresponding properties are listed in Table 1. The VSG model design was for demonstration purposes and was not intended to precisely simulate the actual VSG found in human VFs.

**Table 1.** Synthetic Vocal Fold Model Layer Compositions

Model layer	Target tensile modulus (kPa)	UV Electro mixing ratio	Extruder A (%)	Extruder B (%)
Body	12	1:3.21	100	0
Ligament	2	1:8.23	14.98	85.02
SLP EPI	1.5	1:9.12	0	100
SLP VSG	1.5–4.5	1:9.12–1:5.81	0–55.92	100–44.08

“SLP EPI” and “SLP VSG” correspond to the SLP layer of the EPI and VSG models, respectively. The SLP VSG ratios were chosen so that the design stiffness would decrease linearly in the superior direction by a factor of 3. Mixing ratios are (base+catalyst):(thinner) by weight. The relative amounts of material deposited by extruders A and B required to achieve the desired mixing ratios are also shown.

EPI, epithelium; SLP, superficial lamina propria; UV, ultraviolet; VSG, vertical stiffness gradient.

The approximate print times for the EPI and VSG VF models were 47 and 44 min, respectively. After printing and curing, the models were adhered to polylactic acid 3D-printed mounts using Sil-Poxy, with threads inserted into the ligament layers and an epithelial layer added following the practice of Murray and Thomson^2,3^ and as detailed by Young.^27^ As described by Murray and Thomson,^2,3^ applying tension to the threads enabled simulation of the anisotropic effects of the human VF ligament.

To explore repeatability, sets of models were printed on different days, with eight EPI models (i.e., four model pairs) on day 1 and eight VSG models on day 2. The UV Electro was mixed on day 1 and used for both days but stored in a dark cabinet between days to prevent curing. The process was repeated 1 week later with a new UV Electro mixture, with eight VSG models printed on day 7 and eight EPI models on day 8.

Six 10 mm cubes were printed to measure the tensile properties of the materials used to print the VF models, three of the extruder A material and three of extruder B, which correspond to the materials of the body and SLP layers, respectively. Cubes were printed on the same days as the corresponding VF models using the same print settings and silicone mixtures. The cube tensile tests were conducted ∼24 h after being printed.

In addition to material and geometric properties, characteristics that define a successful synthetic VF model are the abilities to withstand vigorous flow-induced vibration and to vibrate within the correct ranges of onset pressure (minimum air pressure required to initiate vibration), frequency, and glottal width amplitude. Pressure, flow, and high-speed video data of model vibration were acquired to evaluate model response. See the study by Young^27^ for experimental setup and procedure details, but to summarize, for each test, two models in a full larynx configuration were mounted at the end of a 33-cm-long, 2.4-cm-diameter tube attached to a rigid plenum connected to a compressed air source.

The air flow was gradually increased until a sudden audible vibration noise indicated that vibration had commenced and the onset pressure was recorded. The air flow was then decreased until vibration stopped. This process was repeated two more times, and the average onset pressure was calculated. High-speed videos were captured at 20% above onset pressure and analyzed to calculate vibration frequency and glottal width amplitude. Two sets of thread tension conditions were tested: one in which tension was applied via two 21.5 g weights hung from threads tied together on the anterior and posterior ends (one weight on each side), and the other in which no weight was applied.

## Results

### Process validation

#### Material stiffness

**FIG. 4.**
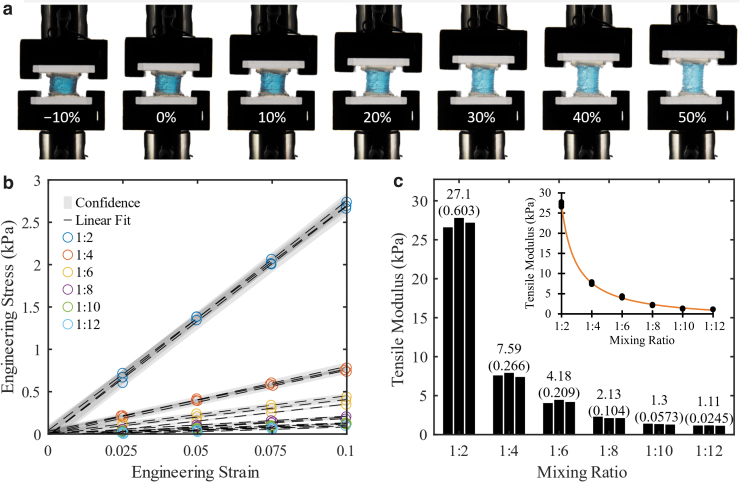
**(a)** Images of cube tensile testing between −10% and +50% strain. **(b)** Stress versus strain for cubes with mixing ratios as depicted in the legend (three cubes per ratio). *Symbols* denote data points, and *dashed lines* are linear curve fits. The 95% confidence interval was calculated using twice the standard deviation. **(c)** Corresponding tensile modulus values (slopes of linear curve fits) versus mixing ratio. Bar chart labels are averages, with standard deviations in *parentheses*. *Inset* shows rational polynomial curve fit to the modulus versus mixing ratio data as given by the equation $y=\left(-1.554x+28.73\right)∕\left(x-1.056\right)$, where *y* denotes the modulus in kPa and *x* denotes the thinner ratio (e.g., 1:x).

Stress–strain data from the initial stiffness tests of cubes printed with mixing ratios of 1:2 to 1:12 are shown in Figure 4. The data are linear and show the capability to print UV Electro silicone within a tensile elasticity modulus range of 1.11 to 27.1 kPa. Attempts to print stiffer UV Electro using a ratio of 1:1 were unsuccessful due to the high viscosity of the mixture, and curing of silicones with ratios >1:12 was unreliable. By way of comparison, the layer modulus values of the synthetic EPI VF model of Murray and Thomson,^2^ excluding the epithelium layer, were 0.2, 1.6, and 11.8 kPa for the SLP, ligament, and body layers, respectively. Only the 0.2 kPa SLP layer does not fall within the present range. Other VF model studies report SLP and/or cover layer stiffness values of 0.91 kPa,^26^ 1.5 and 2.94 kPa,^32^ 4.1 kPa,^33^ and 2 kPa.^34^

#### Spatial stiffness variations

**FIG. 5.**
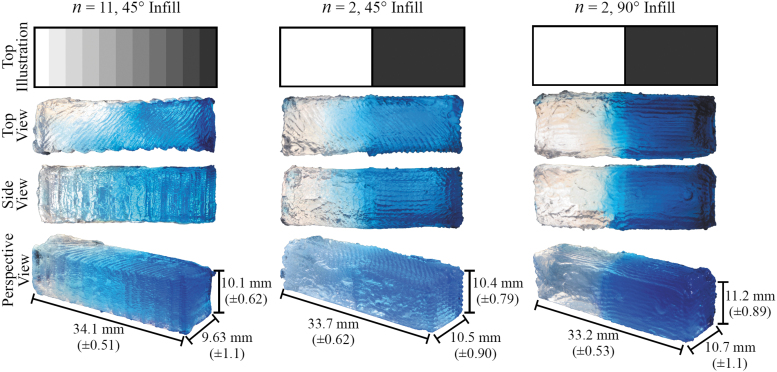
Illustrations (*top row*) and images from different views (*bottom three rows*) of printed cuboids with *n* = 11 and 45° infill orientation (*left column*), *n* = 2 and 45° orientation (*center*), and *n* = 2 and 90° orientation (*right*). Cuboids were printed in the orientation as shown in the perspective view, with the top face being printed as the last layer. Dimensions are given with average error shown in *parentheses*.

Illustrations and images of cuboids with material variations are shown in Figure 5. The variations can be visualized by color from clear (least stiff; extruder B with 1:9.12 ratio) to blue (stiffest; extruder A with 1:3.21 ratio). General shapes were as desired, although additional work is needed to improve print quality. The top views show ridges that match the orientations of the infill-pattern angles. Deformities near the cuboid ends and at the interfaces between material sections are evident and are attributed to over-extrusion as the needles briefly came to a stop and the extruder retracted in preparation for material transition. Another likely source of geometric inaccuracy is the difference in liquid silicone viscosity due to different amounts of thinner. Other adverse printing artifacts may be due to factors such as accumulated over-extrusion, needle retraction effects, and fluidic motion caused by the needles traversing through the support matrix.

**FIG. 6.**
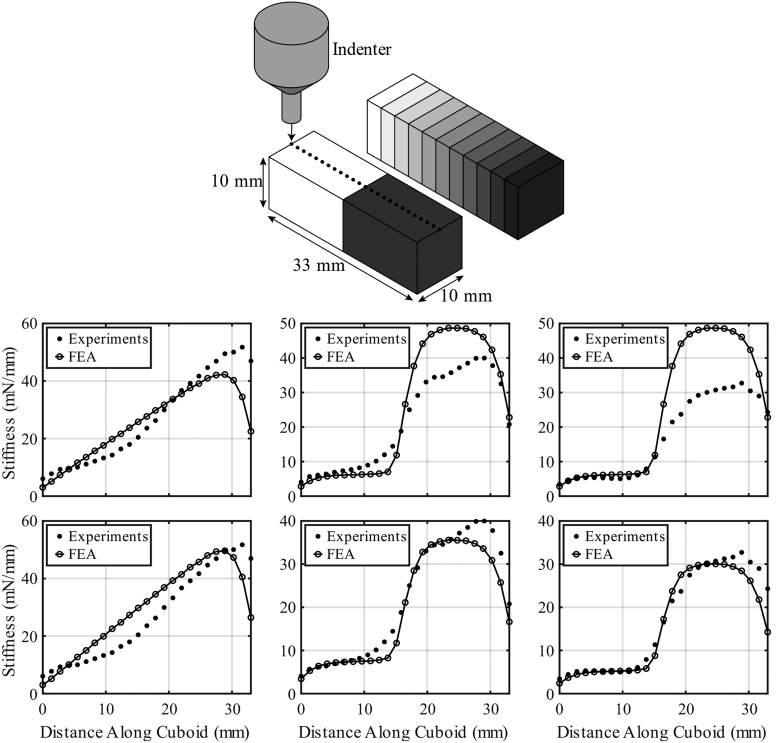
(*Top illustrations*): Illustration of compression testing of cuboids with *n* = 2 (*lower left*) and *n* = 11 (*upper right*) material sections. *Dots* denote the 25 testing locations. (*Bottom* charts): Pre-tuned (*top row*) and post-tuned (*bottom row*) stiffness versus distance data from experiments and FEA with *n* = 11, 45° orientation (*left column*); *n* = 2, 45° orientation (*middle column*); and *n* = 2, 90° orientation (*right column*). Stiffnesses were calculated using linear fits to force versus plunge depth data. The *clear-to-blue* regions pictured in Figure 5 correspond to 0–33 mm, respectively, in these plots. FEA, finite element analysis.

The experimental and FE results are shown in Figure 6. The correlations between the trends of the experimental and FE results are encouraging. For the initial FE analyses, each section's material model was assigned a uniform modulus value that corresponded to the target modulus values of the printed cuboids. The initial FE stiffness results were compared with the experimental results, the FE modulus values were tuned to more closely match the experimental data, and the FE analyses were repeated (see Supplementary Information for further details). Given variabilities inherent in processing and printing these ultrasoft silicones with high concentrations of silicone thinner, the need for material tuning is not surprising. Figure 6 shows pre- and post-tuned data.

The stiffness gradients are evident in both experimental and FE results. The *n* = 2 cuboid results demonstrate the ability to transition between the two materials and show that print quality is affected by infill orientation. Less color bleeding between the two material sections can be seen in the *n* = 2, 90° infill orientation images in Figure 5, and the data in Figure 6 show that the stiffness of the 90° cuboid follows that of the FE model slightly more closely between the two material sections. This has implications to printing complex geometries, such as VF models, and future work will be required to study the effects of infill orientation to optimize print quality.

### VF model printing and testing

#### Geometry

**FIG. 7.**
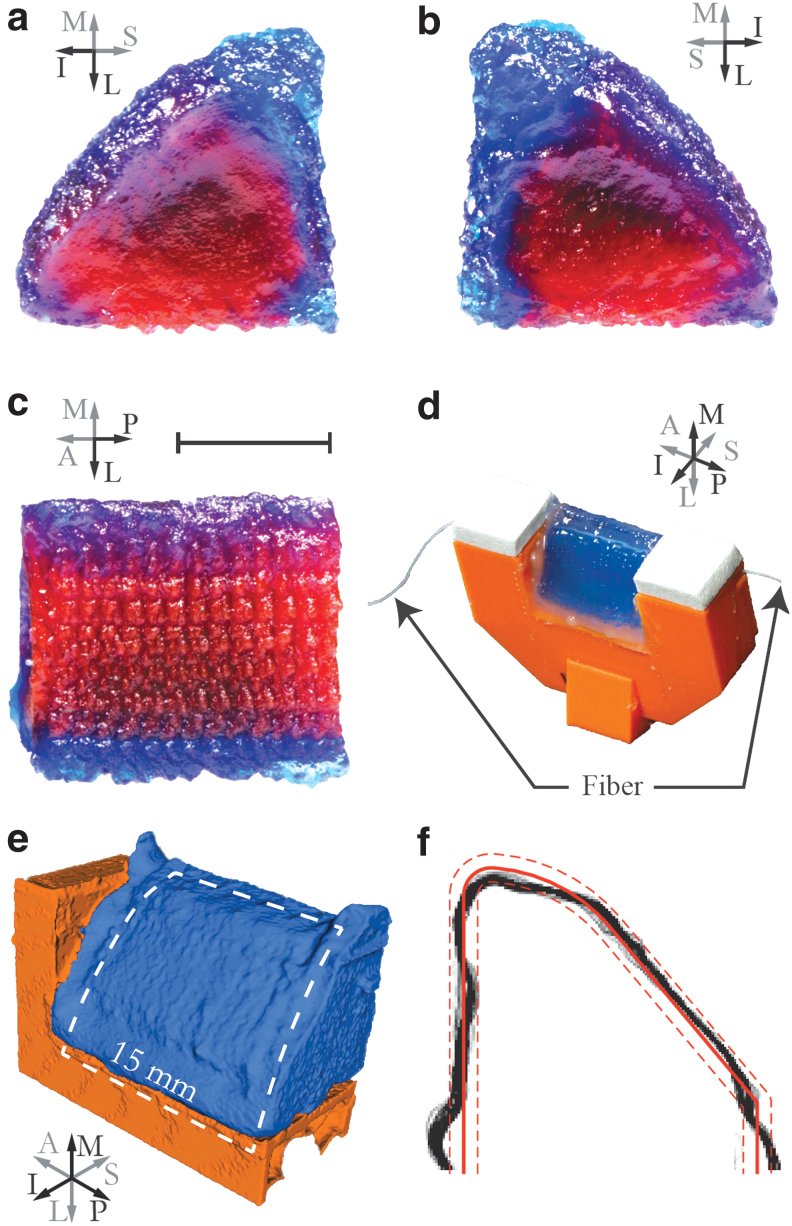
**(a–c)** Printed EPI model with *red and blue dye* corresponding to the body and SLP layers, respectively (no epithelial layer). About 2 mm of the anterior and posterior ends of the model have been cut away for improved layer visibility. Scale bar is ∼5 mm. **(d)** A different printed model, mounted, with fiber inserted, and inclined for pouring of epithelial layer. Models were printed with an anterior–posterior build direction. **(e)** Micro-CT scan of mounted EPI model (*blue*) and mount (*orange*) (the near side of the mount was not segmented to enable visibility of the VF model). The *white dashed line* denotes the approximate location of the inner 15 mm region of the VF used to create the image in **(f)**. **(f)** Superimposed perimeters from more than 200 sections (*black*) with design perimeter (*solid red*) and ±0.5 mm offset (*dashed red*) for reference. The *red perimeter* was defined using the same scale as the *black perimeter*, but positioned by minimizing the average error. CT, computed tomography.

Images of printed EPI models and data from micro-computed tomography (CT) images (72 μm resolution; Quantum GX, Hopkinton, MA) of a printed EPI model that had undergone vibration testing are shown in Figure 7. Interior layers are evident in the cross sections but could not be differentiated in the micro-CT data. More than 200 frontal plane images from a 15-mm anterior–posterior section of the micro-CT-imaged model were imported and analyzed (glued end regions were excluded). The outer perimeters were identified using a custom MATLAB script,^27^ superimposed on each other, registered, and overlaid with a stencil of the desired geometry as shown.

The thickness of the black perimeter represents the outer geometry consistency in the anterior–posterior direction, and the distance between the black perimeter and red stencil overlay represents the error at a given point on the VF perimeter. Much of the black perimeter is <0.5 mm thick and falls within the ±0.5 mm offset bands. The average error of the perimeter was calculated to be ±0.29 mm (quantified by averaging the distance of each black pixel in each frontal plane image to the nearest point on the perimeter of the stencil).

#### Material properties

The tensile modulus data of the cubes representing the body and SLP layers are provided in Table 2. Additionally, data from previous cast EPI VF models^2^ and embedded 3D-printed EPI VF models^26^ are included for reference. The stiffnesses of the body and SLP EPI layers of the 3D-printed VF models were comparable to, but somewhat greater than desired. Week 1 layers were 1.3 and 0.44 kPa stiffer than desired (by 10.9% and 29.5%, respectively) and week 2 layers were 3.8 and 0.95 kPa stiffer than desired (by 31.8% and 63.3%, respectively). Possible reasons for these variations may include inherent 3D printing variability, small differences in material mixing ratios, amounts of glue used when attaching the cubes to the tensile mounting plates, alignment of the cube specimens in the mounting fixtures during tensile testing, and fluctuations in room temperature and humidity during material preparation and testing. Experience has shown that ultrasoft silicones such as these are very sensitive to such processing parameters, so the variations seen in Table 2 are not unexpected.

**Table 2.** Modulus Values for Current Printed Vocal Fold Models (First Three Columns) and Previous Cast (Fourth Column) and Embedded Three-Dimensional-Printed (Last Column) Models

	Tensile modulus (kPa)				
	Target values	Week 1 models	Week 2 models	Previous cast EPI VF model	Previous embedded 3D-printed EPI VF model
Body	12	13.3 (±1.51)	15.8 (±1.78)	11.8^a^	12.9^b^
SLP EPI	1.5	1.94 (±0.131)	2.45 (±0.152)	0.200^a^	0.910^b^

Column 1 lists the target tensile modulus values for this study. Columns 2 and 3 are average tensile moduli of the printed cubes, with the standard deviations in parentheses.

^a^ Murray and Thomson.^2^

^b^ Greenwood and Thomson.^26^

3D, three-dimensional; VF, vocal fold.

#### Vibratory properties

**FIG. 8.**
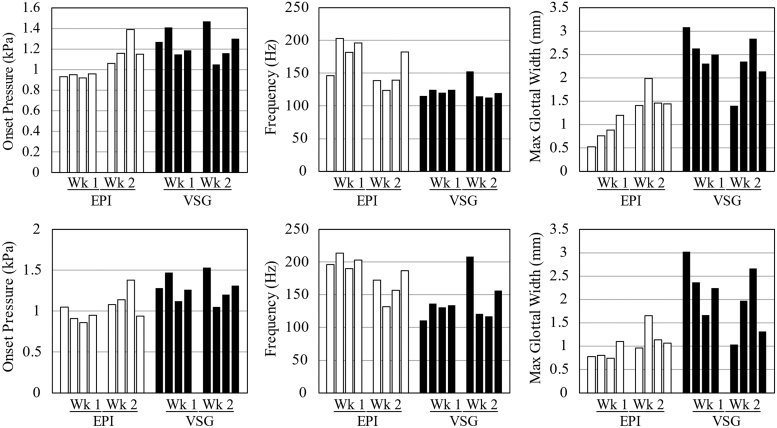
Onset pressure, frequency, and maximum glottal width for all EPI and VSG VF models in non-tension (*top*) and tension (*bottom*) from weeks 1 and 2. Wk, week.

Vibration test data for the EPI and VSG models, with and without tension, are listed in Table 3. Corresponding data for the previous cast EPI VF models,^2,3^ single-material 3D-printed VF models,^24^ embedded 3D-printed models,^26^ as well as human *in vivo* VFs are also included for reference. Additionally, the onset pressure, maximum glottal width, and frequency data for each of the printed EPI and VSG VF models in tension and non-tension are plotted in Figure 8.

**Table 3.** Printed Vocal Fold Model Vibration Test Data

Model	Onset pressure (kPa)	Vibration frequency (Hz)	Maximum glottal width (mm)
3D-printed EPI model, no tension	1.07 (±0.16)	164 (±30.16)	1.21 (±0.47)
3D-printed EPI model, tension	1.04 (±0.17)	181 (±26.70)	1.03 (±0.30)
3D-printed VSG model, no tension	1.25 (±0.14)	123 (±12.71)	2.40 (±0.51)
3D-printed VSG model, tension	1.28 (±0.16)	139 (±31.21)	2.04 (±0.67)
Cast model, no tension^a^	0.37	86^b^	2.4^b^
Cast model, tension^a^	0.27	102^b^	1.4^b^
Single-material 3D-printed model, no tension^c^	3.8–3.9	181–214	—
Embedded 3D-printed model, tension^d^	0.82–1.24	125–180	0.91–3.2
Human	0.29–0.49^e^	100–136 (male)^e^ 189–224 (female)^e^	2^f^

Results from weeks 1 and 2 were combined and averaged, with standard deviations in parentheses. Data from previous studies are included for comparison.

^a^ Murray and Thomson.^2,3^

^b^ Values are approximated from Murray and Thomson^3^ plots at 120% onset pressure.

^c^ Romero *et al.*^24^

^d^ Greenwood and Thomson.^26^

^e^ Baken and Orlikoff.^35^

^f^ Schuberth *et al.*^36^

The onset pressures for printed models ranged from 0.86 to 1.53 kPa. These are higher than the range of human onset pressures reported by Baken and Orlikoff,^35^ but still within the range of human lung pressure during voicing (at least as high as 2.72 kPa at loud intensities^35^). Additionally, the onset pressures of the models in this study are lower than those of the single-material 3D-printed models (3.8–3.9 kPa)^24^ and of a cast VF model based on magnetic resonance imaging data reported by Murray and Thomson (1.68 kPa).^3^ The frequencies and maximum glottal widths of the printed models in this study are comparable to those of human VFs, as shown in Table 3.

The finding that the VSG models had lower frequencies and higher glottal widths than the EPI models is unexpected as the stiffer material in the VSG SLP layer would be expected to increase the frequency and decrease the glottal width. It is possible that this may have been caused by resonance effects due to acoustic coupling in the test setup, although the effects due to printing imprecision or errors cannot be ruled out, and further exploration is needed.

Two-tailed Student's *t*-tests were performed to determine statistical significance between weeks 1 versus 2, tension versus non-tension, and EPI versus VSG models. The primary conclusions are as follows (see Supplementary Information for details). The EPI and VSG models were statistically different, with all *p*-values <0.05. The vibration data seem to exhibit difference in trends in frequency and glottal width between tensioned and non-tensioned models, but the differences were not statistically different. Nine of the 12 *p*-values comparing weeks 1 and 2 were not statistically different; this is encouraging from the standpoint of process repeatability, although research to improve material and printing consistency is desirable.

## Conclusions

The ability to three-dimensionally print ultrasoft silicone with functional stiffness gradients, with the specific application of printing multilayer VF models for voice production research, has been demonstrated. Silicone samples were printed within a tensile elastic modulus range of 1.11–27.1 kPa, a range that is ultrasoft on the low end and suitable for synthetic VF modeling. Cuboid and VF-shaped prints with stiffness variations were presented. The cuboid specimens exhibited stiffness variations that were in reasonable agreement with FE simulations.

The VF models vibrated with flow and vibratory characteristics comparable to previous synthetic VF models. These results demonstrate the potential for this method to constitute a viable alternative to casting for creating synthetic VF models. It is anticipated that the methods described herein may be useful for other applications involving soft materials with stiffness variations, including other biomechanical systems. It is also worth considering that the present dual extruder design concept may be extended to enable the printing of other material property gradients, such as color, thermal conductivity, and optical absorptivity and reflectivity.

Several areas for improvement and future study are here listed. These include improving spatial resolution and print quality by refining hardware setup and print settings; exploration of the use of other silicones (e.g., the Ecoflex family of addition-cure silicones; Smooth-On, Inc.); further development of custom slicer software parameters and path generation capabilities; redesign of dual needle setup using microfluidics; further investigation of printing and material preparation repeatability; exploration of effects of infill orientation pattern on print output; and identifying a nonintrusive method of evaluating the accuracy of printed layer structure (e.g., adding micro-CT-traceable particles to the silicone in one extruder).

Regarding VF modeling, as discussed in the Introduction section one of the limitations of casting VF models is the relatively high rate of failure; this is due to the difficulty of demolding ultrasoft silicones. Because the 3D ultrasoft silicone printing methodology discussed herein is a relatively new technology, the yield of three-dimensionally printed VF models is not yet better than that of cast models. It is hoped that further process refinements will result in improved yield.

## Supplementary Material

Supplemental data
