# Supplementary material for: Three-Dimensional Printing of Ultrasoft Silicone with a Functional Stiffness Gradient
Source: 3D Print Addit Manuf. 2024 Apr 16;11(2):435–45. doi: 10.1089/3dp.2022.0218 (PMC11057526; doi:10.1089/3dp.2022.0218)
Supplement: Supplemental data [file Supp_DataS1.pdf]

Supplemental Information: Three-dimensional printing of ultra-soft silicone with a functional stiffness gradient

Clayton A. Young, MeiLi O'Bannon, and Scott L. Thomson*

Mechanical Engineering, Brigham Young University, Provo, Utah 84602, USA

3D Printer Slicing Software

Most fused filament fabrication (FFF) printing slicing software settings are specific to printing with thermoplastics; however, Romero et al.[24] and Greenwood et al.[25] successfully refined the settings of an open source slicer (Cura) to print with silicone. Several slicers can also "slice" multi-part CAD models and assign different colors to different parts. While this multi-color software function can be implemented for multi-material models, in practice the results have been poor when applied to multi-material silicone 3D printing.[28] The limitations of current FFF slicing software and the advantages of a custom multi-material 3D printing slicer developed for this study are here discussed.

The gel-like support matrix used in this study has thixotropic properties that allow the support matrix to hold the print in place; however, during printing, deposited silicone is susceptible to flow caused by the extrusion needles traversing through the support matrix. This flow can cause unwanted deformities in the final print. It is desirable, therefore, for the slicing software to minimize needle movement in the support matrix, especially movement across previously-printed layers. Two examples of unnecessary needle movement that may cause unwanted flow are material changes and 3D-print infill-patterns.

Needle movement during material changes is the first example of unwanted flow. In standard multi-color FFF 3D printing, the 3D printer must be able to switch from one material to

another. This is often done by either retracting the current material from the extruder and then priming the new material in the same extruder using a purge tower, or by switching between multiple extruders with different materials. Material changes in standard multi-color FFF 3D printing are time consuming, and as a result, slicers are typically designed to minimize the number of material changes. While this may save time, it requires a significant amount of travel movement where no material is being deposited. Because FFF 3D printing with thermoplastics is not susceptible to unwanted flow, the additional travel movement required to minimize material changes is inconsequential. However, for silicone 3D printing, the unwanted flow caused by excess travel due to material changes can cause unwanted deformities in the final print. Two infill-pattern approaches are shown in Fig. S.1. Figure S.1a illustrates an approach in which the extruder prints all of one material in a layer before switching to the next material, whereas Fig. S.1b shows the extruder moving from left to right while changing materials between each section to minimize travel.

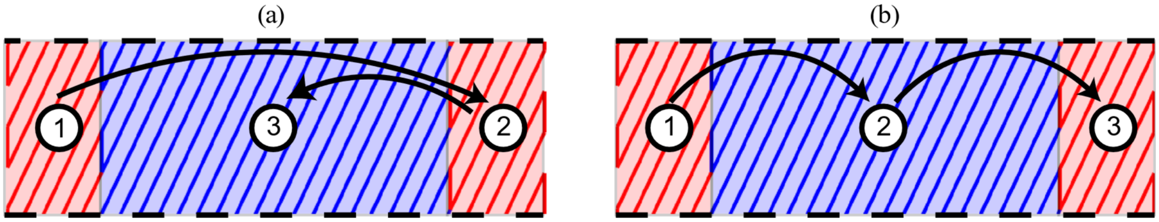

FIG. S.1. Print order for sections of a multi-material layer. Each color represents a different material. (a) All of one material is printed before switching to the next material. (b) The layer is printed from left to right continuously to minimize travel movement.

Excess needle movement caused by infill-patterns is the second example of unwanted flow. Standard 3D-printing slicing software typically offers a variety of print infill-patterns that can be selected. For single-material 3D-printed synthetic vocal fold models, the simple rectilinear pattern with 45° infill-orientation, as shown in Fig. S.1, was found to yield the best printing results;[24]

however, these same results did not translate well to multi-material 3D printing.[28] In the latter case, the infill-pattern is applied to each material section as shown in Fig. S.2a. This causes the needles to turn around at each material section interface, resulting in uncured support matrix becoming trapped between material sections and separation between sections as shown in Fig. S.3. To enable better adhesion between material sections, a continuous infill-pattern across sections could be utilized as shown in Fig. S.2b. While this continuous infill-pattern relies heavily on accurate material changes when crossing each material section interface, it could decrease unwanted flow due to excess travel movement and achieve better adhesion between sections.

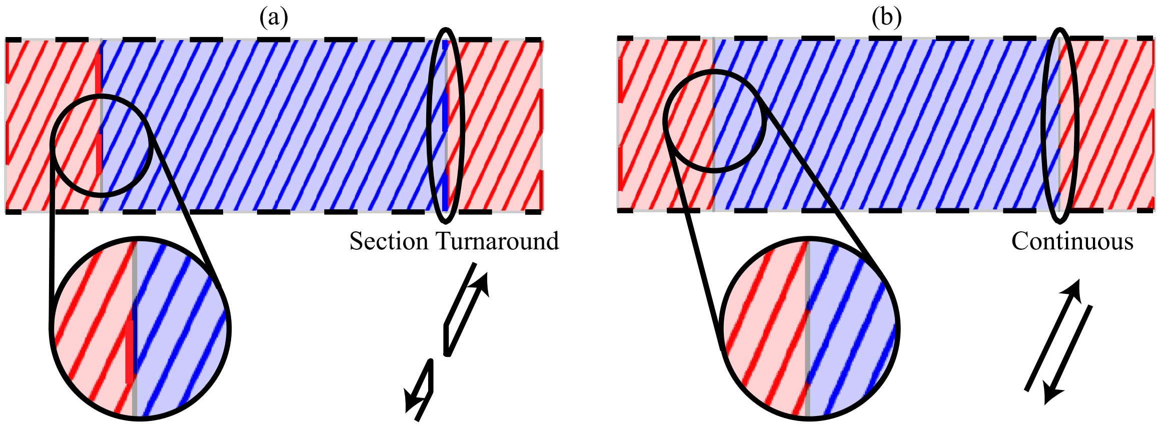

FIG. S.2. Two infill-patterns. (a) Rectilinear pattern with 45° infill orientation applied to each material section causing the needles to turn around and create separation at each material section. (b) Rectilinear infill-pattern applied across the entire print layer creating a continuous infill-pattern with material switching at the interface of different sections.

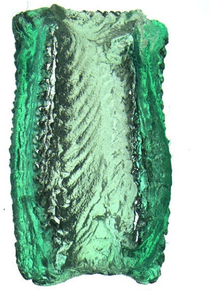

FIG. S.3. Image of the first five print layers of a multi-material print with the rectilinear pattern with 45° infill orientation and section turnaround as shown in Fig. S.2a.

The custom slicing software was designed to overcome the challenges of unwanted flow. The software optimized the print order to reduce travel movement (Fig. S.1b) and achieve a continuous rectilinear infill-pattern (Fig. S.2b). The general fabrication overview (Fig. S.4) is described in detail as follows. First the user created a computer-generated model, where each part of the model corresponded to a different material property. An .stl file of each part of the computer-generated model was imported into the custom slicing software, and the material property of each part was assigned in units of kPa. The slicing software then converted the material properties for each part from kPa to a ratio of UV silicone [(base + catalyst):(thinner)] using the material model described in Young.[29] Using the UV silicone ratio in each extruder, the slicing software then determined the ratio of extruder A and extruder B that was needed for each part of the print.

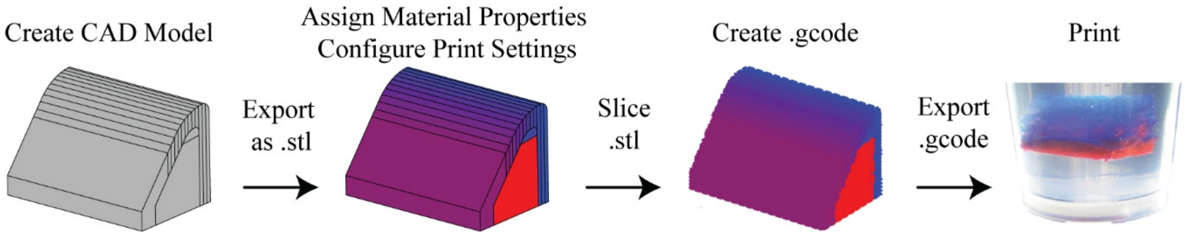

FIG. S.4. Illustration of model fabrication process.

The slicing software then "sliced" the print layer-by-layer creating *x*-, *y*-, and *z*-translation g-code commands for the 3D printer. The layer height was equal to the needle inner diameter (0.21 mm), and the path width of the continuous rectilinear print pattern was equal to a multiple of the needle inner diameter and the percent infill (60%). Other settings that were implemented during the slicing phase of the software include print speed, extruder retraction distance, infill angle, syringe diameter, needle outer diameter, and needle offset.

After the print was "sliced," the part in which each path was located was determined, and the corresponding ratio of extruder A and extruder B was assigned. This ratio was used to calculate the A- and B-extruder g-code commands. Additionally, each path was checked to see if a material change was required, and necessary g-code was added accordingly. Finally, the g-code for each layer was compiled and exported for the entire print.

The software provided a layer-by-layer animation of the print as shown in Fig. S.5. The animation rendering was color-coded relative to material stiffness for visualization and validation, allowing the user to quickly iterate through print settings and observe their effects before finalizing the g-code. The MATLAB script for the multi-material slicing software is included in Young.[29]

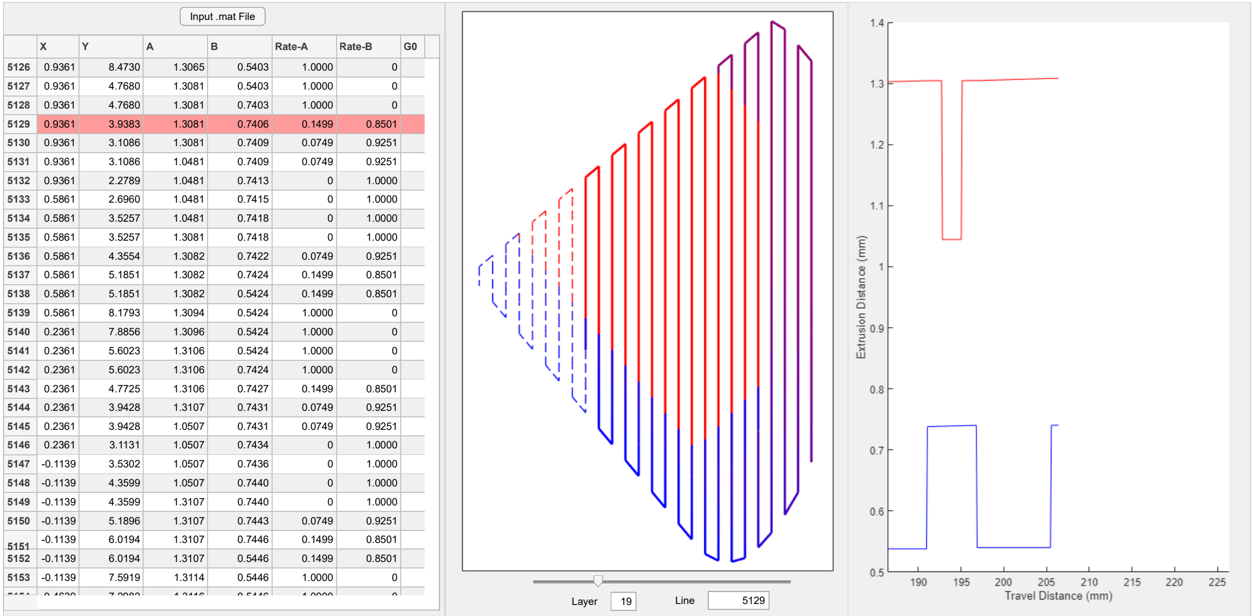

FIG. S.5. Image of the slicing software graphical user interface showing the g-code output (left panel), layer-by-layer animation (center panel), and extruder values (right panel). The interface allows the user to easily navigate between layers and g-code lines to verify correct g-code output.

Finite Element Model

It was expected that compression testing near the cuboid edges as well as near interfaces of material sections with different stiffnesses would affect compression results. Therefore, for comparison and to confirm the effects of edges and material interfaces on the stiffness profiles, finite element analysis (FEA) of each cuboid compression test was performed. An FEA model of the 10×10×33 mm cuboid was created using ANSYS ADPL (Canonsburg, PA, see Young[29] for code). Each material section of the cuboid was modeled using 1 mm high-order SOLID187 3D elements with a bonded boundary condition between each material section. The section materials were modeled using a linear elastic material with a Poisson's ratio of 0.49, and the bottom of the cuboid was fixed in all directions. After the initial meshing, the elements in a hemisphere around the region of indentation were refined to improve the FEA results, with the refinement increasing closer to the contact area as shown in Fig. S.6. The first refinement of the elements was within a 9.6 mm diameter sphere of the compression testing location. The second refinement was within a smaller hemisphere with a diameter 6.4 mm. The last refinement was on the surface of the cuboid, using the same refinement settings, to provide a high density of nodes in a circular pattern with a 3.4 mm diameter.

The initial FEA analyses modeled the indenter as a cylinder and contact pairing with the cuboid. An alternative approach was tested in which the indenter was modeled as a 3 mm diameter circle inscribed on the top of the cuboid with the center being located at the desired testing location. In both approaches, the cylinder or circle was displaced 2 mm into the cuboid over 40 load steps (i.e., 0.05 mm penetration at each load step) and fixed in all other remaining dimensions. The differences in initial cuboid stiffness results were found to be negligible; however, the

computational cost of the cylinder method was higher. Therefore, the final FEA analyses were completed using an inscribed circle rather than a cylinder.

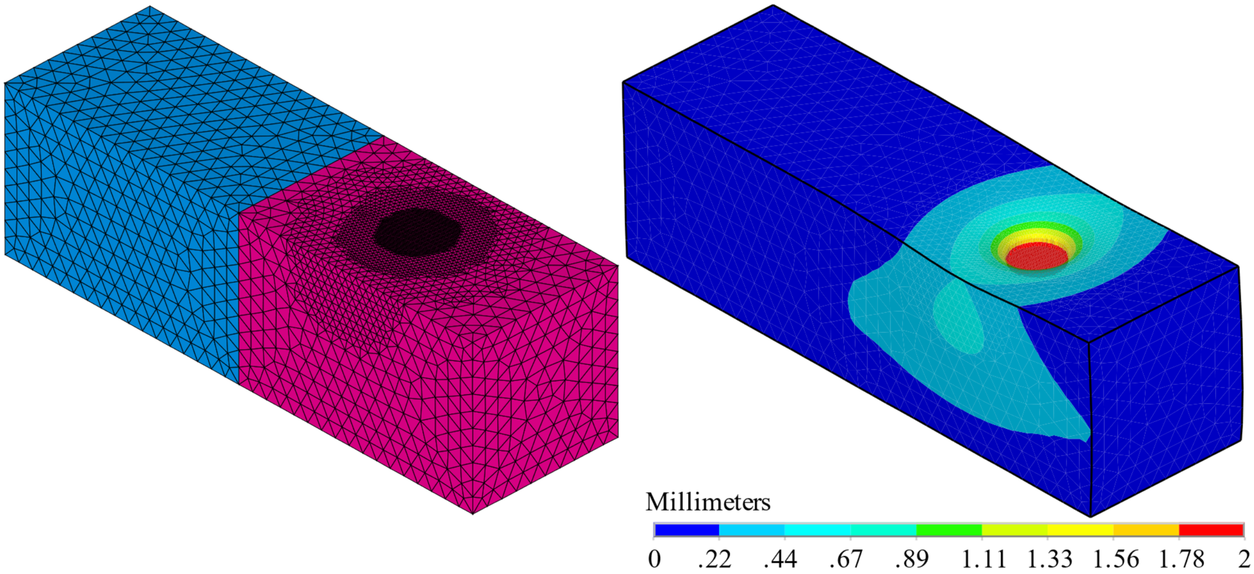

FIG. S.6. (Left) Perspective view of the meshed $n = 2$ cuboid. Refined circular mesh area is located where the indenter is plunged into the cuboid in this instance. (Right) Perspective view of the deformed cuboid with 2 mm indentation. The same meshing and indentation procedure was followed the for the $n = 11$ cuboid.

The initial FEA analyses were solved with *nlgeom* activated in ANSYS to account for large deflections; however, above load step 17 (0.85 mm) the solution frequently became non-convergent. Of the load steps that did solve, the cuboid stiffnesses with *nlgeom* activated and deactivated for the $n = 2$ and $n = 11$ cuboids were a maximum of 3.62% and 3.83% different, respectively, with averages of 1.44% and 1.35%, respectively. With the relatively small average differences between *nlgeom* activated and deactivated, as well as the linearity of the experimental force-displacement data shown in Young,[29] the material for the FEA model was assumed to be linear-elastic and the final FEA analyses were completed with *nlgeom* deactivated.

For the initial FEA analyses, each sections' material model was assigned a uniform modulus value that corresponded to the target modulus values of the printed cuboids (i.e., 1.5 kPa and 12 kPa for the two sections of the $n = 2$ cuboid, and 1.50 kPa, 2.55 kPa, 3.60 kPa, 4.65 kPa, 5.70 kPa, 6.75 kPa, 7.80 kPa, 8.85 kPa, 9.90 kPa, 10.95 kPa, and 12 kPa for the 11 sections of the $n = 11$ cuboid). The FEA stiffness results were then analyzed and compared to the experimental results. The FEA modulus values were then tuned to adjust the stiffness results to more closely match the experimental compression data and the FEA analyses were repeated. The modulus values were tuned by scaling the initial FEA modulus input values by the ratio of the desired FEA stiffness over the actual FEA stiffness at points near the ends of the cuboids. For the $n = 11$ cuboid, the 4th and 22nd data points were selected, and for the $n = 2$ cuboids, the 4th and 18th data points were selected. The primary purpose behind performing FEA analyses was to validate the trends (i.e., stiffness gradients, edge effects, and material interfaces) that were present in the experimental data, therefore, stiffness tuning was deemed to be an acceptable approach to better compare the trends. Final FEA modulus values are listed in Table S.1, and a comparison between pre- and post-tuned stiffness plots are shown in Fig. 6.

Table S.1. Post-tuned lower and upper modulus values for the cuboid FEA models. The initial (pre-tuned) lower and upper modulus values were 1.5 and 12 kPa, respectively.

Cuboid Model	Lower Modulus (kPa)	Upper Modulus (kPa)
$n = 11$, 45°	2.24	13.4
$n = 2$, 45°	1.80	8.75
$n = 2$, 90°	1.26	7.41

Statistical Analysis

Two-tailed Student t-tests were performed to determine statistical significance between week one vs. week two, tension vs. non-tension, and EPI vs. VSG models using the three vibration metrics of onset pressure, frequency, and maximum glottal width. The results are listed in Table S.2. Each metric indicates that the EPI and VSG models were statistically different, with all P-values less than 0.05. The vibration data seem to suggest differences in trends in vibration frequency and maximum glottal width between tensioned and non-tensioned synthetic VF models; however, Table S.2 shows that the differences were not statistically significant. Furthermore, the high statistical P-values for onset pressure for the EPI and VSG models are notably indicative of an absence of statistical difference between tensioned and non-tensioned VF models. Care was taken to follow similar fabrication and vibration testing procedures of models from weeks one and two. Nine of the twelve P-values comparing week one and week two are not statistically significant. This is encouraging from the standpoint of process repeatability, although research to further improve consistency is certainly desirable.

Table S.2: Statistical P-values of two-tailed Student t-tests for all vibration testing values and VF model categories. Asterisk denotes P-value less than 0.05.

Test Comparison	Statistical P-value		
	Onset Pressure	Vibration Frequency	Maximum Glottal Width
EPI vs VSG Non-tension	0.029*	0.006*	0.0002*
EPI vs VSG Tension	0.012*	0.012*	0.003*
EPI Tension vs Non-tension	0.755	0.243	0.388
VSG Tension vs Non-tension	0.723	0.203	0.239
EPI Week 1 vs Week 2 Non-tension	0.038*	0.094	0.009*
EPI Week 1 vs Week 2 Tension	0.127	0.040*	0.102
VSG Week 1 vs Week 2 Non-tension	0.929	0.731	0.249
VSG Week 1 vs Week 2 Tension	0.939	0.381	0.253
